# Environmental Factors Can Influence Mitochondrial Inheritance in the *Saccharomyces* Yeast Hybrids

**DOI:** 10.1371/journal.pone.0169953

**Published:** 2017-01-12

**Authors:** Yu-Yi Hsu, Jui-Yu Chou

**Affiliations:** Department of Biology, National Changhua University of Education, Changhua, Taiwan, R.O.C.; Virginia Tech Virginia, UNITED STATES

## Abstract

Mitochondria play a critical role in the generation of metabolic energy and are crucial for eukaryotic cell survival and proliferation. In most sexual eukaryotes, mitochondrial DNA (mtDNA) is inherited from only one parent in non-Mendelian inheritance in contrast to the inheritance of nuclear DNA. The model organism *Saccharomyces cerevisiae* is commonly used to study mitochondrial biology. It has two mating types: MATa and MATα. Previous studies have suggested that the mtDNA inheritance patterns in hybrid diploid cells depend on the genetic background of parental strains. However, the underlying mechanisms remain unclear. To elucidate the mechanisms, we examined the effects of environmental factors on the mtDNA inheritance patterns in hybrids obtained by crossing *S*. *cerevisiae* with its close relative *S*. *paradoxus*. The results demonstrated that environmental factors can influence mtDNA transmission in hybrid diploids, and that the inheritance patterns are strain dependent. The fitness competition assay results showed that the fitness differences can explain the mtDNA inheritance patterns under specific conditions. However, in this study, we found that fitness differences cannot fully be explained by mitochondrial activity in hybrids under stress conditions.

## Introduction

The term “mitochondrion” is derived from the Greek word “mitos,” which means “thread,” and “chondrion,” which means “granule” or “grain-like.” Mitochondria are unusual organelles and are found in most eukaryotic cells. They are commonly referred to as the powerhouse of the cell and are enclosed by two lipid membranes. Mitochondria generate chemical energy called adenosine triphosphate (ATP). These organelles supply all the necessary biological energy of the cell through oxidative phosphorylation [1]. Mitochondria are the metabolic mediator of the cell and are involved in energy conservation and calcium ion homeostasis [2]. Mitochondria also regulate various cellular processes, including proliferation [3] and apoptotic cell death [4]; mediate secondary massager signals to the nucleus [5]; and regulate aging [6–8]. In humans, mitochondrial DNA (mtDNA) mutations can affect health and result in many diseases including diabetes, neurodegenerative disorders, cancers, and cardiac dysfunction [9–15].

Mitochondria are unique organelles because they have their own DNA and ribosomes and can produce their own proteins. The mitochondrial genome is circular and double-stranded, whereas the nuclear genome is linear. The majority of mitochondrial genomes form linear head-to-tail molecules which are replicated by rolling circle mechanism [16–17]. In addition, mtDNA can replicate independent of the cell cycle and nuclear genome replication. Mitochondria are considered unique semi-autonomous cellular organelles because they have their own genome and can produce their own genetic material (mtDNA). In most sexual eukaryotes, mtDNA is inherited from only one parent [18–20]. This inheritance pattern is generally referred to as uniparental inheritance. Uniparental inheritance of mtDNA, as well as chloroplast DNA (cpDNA), has been observed in isogamous organisms such as *Chlamydomonas reinhardtii* [21]. *C*. *reinhardtii* is a single-cell green alga with a diameter of approximately 10 μm that swims with two flagella. *C*. *reinhardtii* has two mating types, mating type plus (mt+) and mating type minus (mt−), rather than female and male. Research has summarized that the preferential digestion of the chloroplast from the mt− parent results in the maternal inheritance of cpDNA. Interestingly, data have shown that the mtDNA from both parents persists in young zygotes. However, only mt− mtDNA is inherited after mitosis, whereas mt+ mtDNA is selectively eliminated from the zygotes and leads to the paternal inheritance of mtDNA [21–23].

In anisogamous organisms (i.e., angiosperms), mtDNA is strictly maternally inherited. Two main mechanisms have been suggested to explain this phenomenon. The mitochondria of the smaller gamete (e.g., sperm) may either fail to enter the larger gamete (e.g., egg). Alternatively, the few mitochondria from the sperm may be injected into the egg but may be degraded by ubiquitination, which involves universal substrate-tagging for proteolysis [24–26]. Nevertheless, the two main mechanisms cannot explain uniparental mtDNA inheritance in isogamous organisms such as *Cryptococcus neoformans*. *C*. *neoformans* is a basidiomycete fungus that is found worldwide and causes disease in humans and animals. It has two mating types: MATa and MATα. The hybrids inherit mtDNA from the MATa parent, and results have suggested that mtDNA from the MATα parent is eliminated soon after mating [27, 28].

It is obvious that the mating type can control mtDNA inheritance in *Cryptococcus* fungus. Therefore, different organisms have different strategies for mitochondrial inheritance. Baker’s yeast, *Saccharomyces cerevisiae*, is also an isogamous organism similar to *C*. *neoformans*. In baker’s yeast, the input frequencies of mtDNA genotypes are equal in zygotes obtained by mating different yeast strains, but most diploid progeny are homoplasmic after no more than 20 generations [29]. It has been reported that one parental mitochondrial genotype is preferentially transmitted into hybrid progeny when *S*. *cerevisiae* is crossed with *S*. *bayanus* [30, 31]. *S*. *cerevisiae* is an excellent model organism to study the cellular and biochemical pathways required for the maintenance of respiratory activity, because it can survive on ATP generated by fermentation [32, 33]. Even when oxygen is available, yeast cells generate ATP primarily by fermentation by using fermentable carbon sources, such as glucose, present in the growth medium. Thus, oxidative phosphorylation and the presence of the mitochondrial genome are dispensable. However, when yeast cells are grown on non-fermentable carbon sources, such as glycerol or ethanol, respiration and the presence of an intact mitochondrial genome are essential. Therefore, mitochondrial function might be associated with different carbon sources. Although the exact mechanism underlying such uniparental mitochondrial inheritance is still unclear, various hypotheses have been proposed. The studies have shown that methylation (such as UV irradiation) or ubiquitination (such as treatment with ammonium chloride) can influence chloroplast inheritance in *C*. *reinhardtii* and mitochondrial behavior in mammals during mating [22, 26, 34]. In addition, UV irradiation and temperature influence the inheritance of mtDNA during sexual mating in *C*. *neoformans* [35]. These results suggest the need for investigating the effects of environmental factors on mtDNA inheritance. Components encoded in both nuclear and mitochondrial genomes are necessary for mitochondrial biogenesis, but the coordination of these genes and the regulation of mitochondrial maintenance are not fully understood. Screening for environmental factors that influence mtDNA inheritance may provide clues to the molecular mechanisms involved. Thus, this study mainly investigated the effect of environmental factors on mtDNA transmission in yeast hybrids produced by crossing two close species. Moreover, this study investigated the influence of the genetic backgrounds of different strains on mtDNA inheritance. Subsequently, the fitness difference between hybrids carrying different mtDNA genotypes was evaluated. Finally, mitochondrial activity was also examined using the triphenyl-tetrazolium-chloride (TTC) assay.

## Materials and Methods

### Yeast strains

*S*. *cerevisiae* strains W303 (*MATa*) and UWOPS83-787.3 (*MATa*) and its close relative *S*. *paradoxus* strains YDG 197 (*MAT*α) and N-17 (*MAT*α) were gifts from Dr. Jun-Yi Leu (Institute of Molecular Biology, Academia Sinica, Taiwan). Yeast cells were stored at −80°C with 15%–20% glycerol. Before the experiment, yeast was streaked onto YPD agar plates containing 1% yeast extract, 2% peptone, 2% glucose, and 2% agar and incubated at 28°C for 2 days.

### Crosses between *S*. *cerevisiae* and *S*. *paradoxus* under different stress conditions

**Fig 1:**
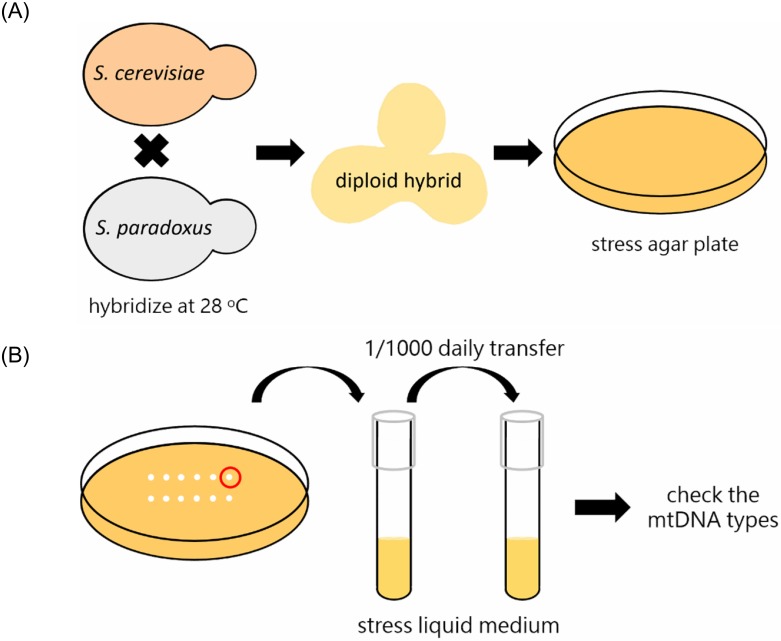
Testing the influence of different stress conditions on mtDNA transmission in hybrids. (A) *S*. *cerevisiae* and *S*. *paradoxus* were refreshed on an YPD agar plate at 28°C overnight before mating experiments. *S*. *cerevisiae* and *S*. *paradoxus* were crossed and incubated for 3 hours at 28°C to form diploid hybrid cells. Hybrids were picked under a dissecting microscope and immediately cultured under different conditions (2% glucose, 5% glucose, glycerol, osmotic pressure, and ubiquitination inhibition). (B) After these hybrids formed colonies on the solid agar plate, all hybrid cells were grown in liquid medium under the respective conditions, and growth was maintained with daily serial transfers (1:1000 dilution). The mtDNA genotypes were identified after approximate 110 generations according to the digestion at the polymorphic sites of *COX3* with the restriction enzymes.

*S*. *cerevisiae* and *S*. *paradoxus* were streaked on YPD agar plates and incubated at 28°C for 1 day and then were refreshed for 3 hours before mating experiments. The mating type a of one species (*S*. *cerevisiae*) were crossed with the α cells of the other species (*S*. *paradoxus*) by mass mating and incubated at 28°C for 3.5–4.5 hours on YPD agar plate to form diploid zygotes. After that, spread mating mixture on YPD agar plate and the zygotes (dumbbell-shaped cells) were isolated by a dissecting microscope (MB-630, MAJOR, Taiwan) during conjugation of two cells and immediately cultured at respective conditions before the first daughter cells bud from the hybrids. After these hybrids formed colonies on a solid agar plate, all hybrid cells were moved into liquid medium under the respective conditions, and growth was maintained with daily serial transfers (1:1000 dilution) (Fig 1). Here we used YPD medium containing 2% glucose, 5% glucose, 0.5 M sodium chloride, or 100 mM NH_4_Cl. Furthermore, yeast extract-peptone-glycerol (YPG) medium was also used to investigate the effects of carbon source. In a specific cross, the resulting hybrids can be considered as biological replicates. The number of biological replicates can be found in Tables 1 and 2. In this study, two technical replicates were tested and only one was shown here.

**Table 1:** Effects of environmental factors on mitochondrial DNA inheritance in hybrids produced by crossing *S*. *cerevisiae* strain W303 with *S*. *paradoxus* strain YDG 197.

Treatment	No. of progeny with mtDNA type from *S*. *cerevisiae*	No. of progeny with mtDNA type from *S*. *paradoxus*	No. of progeny with mtDNA type from both parents	
2% glucose	4	19	15	
5% glucose	21	9	6	p = 7.681x10^-5^, χ2 = 18.948, df = 2 in comparison with those in the 2% glucose condition
glycerol	5	25	5	p = 5.4589x10^-2^, χ2 = 5.8158, df = 2 in comparison with those in the 2% glucose condition
5 M NaCl	20	12	4	p = 9.2563x10^-5^, χ2 = 18.575, df = 2 in comparison with those in the 2% glucose condition
100 mM NH_4_Cl	1	16	16	p = 4.1771x10^-1^, χ2 = 1.7459, df = 2 in comparison with those in the 2% glucose condition

**Table 2:** Effects of environmental factors on mitochondrial DNA inheritance in hybrids produced by crossing *S*. *cerevisiae* strain UWOPS83-787.3 with *S*. *paradoxus* strain N-17.

Treatment	No. of progeny with mtDNA type from *S*. *cerevisiae*	No. of progeny with mtDNA type from *S*. *paradoxus*	No. of progeny with mtDNA type from both parents	
2% glucose	20	2	15	
5% glucose	19	9	7	p = 2.5484x10^-2^, χ2 = 7.3394, df = 2 in comparison with those in the 2% glucose condition
glycerol	34	1	5	p = 1.1917x10^-2^, χ2 = 8.8595, df = 2 in comparison with those in the 2% glucose condition
5 M NaCl	17	11	8	p = 1.362x10^-2^, χ2 = 8.5924, df = 2 in comparison with those in the 2% glucose condition
100 mM NH_4_Cl	9	15	11	p = 6.4751x10^-4^, χ2 = 14.685, df = 2 in comparison with those in the 2% glucose condition

The halo assay is used to identify the mating type of a strain of interest by using tester strains (*bar1*Δ and *sst2*Δ) that are supersensitive to mating pheromones. The strain of interest is patched (or stamped or replica plated) onto a sensitive tester lawn, and the formation of halos on the lawn (because of growth inhibition of the sensitive strain) indicates that the strain is the opposite mating type of the tester. Thus, the a/alpha diploids are not responsive to mating pheromone of either type [36, 37]. After the halo assay, the hybrid cells were also assessed on the plate containing glycerol as the only carbon source to determine whether these selected cells were respiration-competent cells.

### Determining progeny mtDNA genotypes

DNA extraction was performed according to our previous study [38]. The polymerase chain reaction (PCR) mixture was prepared as follows. First, 1 μL of genomic DNA, used as the template; 5 μL of 2 mM dNTP; 1 μL of 10 μM primers flanking mitochondrial *COX3* gene (forward: 5’-TATGCCTTCACCATGACC-3’ and reverse: 5’-TCCAACATGATGTCCAGC-3’); 5 μL of Taq buffer; and 0.25 μL of Taq DNA polymerase was sequentially added to the reaction mixture. The total reaction volume was then adjusted to 50 μL by using sterile ddH_2_O. PCR amplification was performed in a thermocycler (G02, ASTEC, Japan) as follows: (1) initial DNA denaturing at 95°C for 5 minutes, (2) DNA denaturing at 95°C for 1 minute, (3) primer annealing at 50°C for 30 seconds, and (4) DNA elongation at 72°C for 1 minute. Steps 2–4 were circulated 35 times, and the final step of extra elongation was conducted at 72°C for 5 minutes.

Then the restriction enzymes *Hin*dIII and *Bsm*I (GeneMark, Taiwan) was used to identify the mtDNA genotypes, because of the polymorphic sites between *S*. *cerevisiae* and *S*. *paradoxus* (S1 Fig). The PCR product was digested individually with 20 U of the restriction enzymes *Hin*dIII and *Bsm*I at 37°C for 1 hour in 50-μL reaction mixtures. The products were analyzed using 2% agarose gel electrophoresis, and a 1-kb DNA ladder (PU-GDM-201, PURIGO, Taiwan) was used as the DNA marker. The gels were stained with ethidium bromide (fluorescent dye) that binds to DNA and placed on an ultraviolet transilluminator (MS-MUV21-312, Scientific Biotech Corp., Taiwan) to visualize the stained DNA as bright bands. Subsequently, the mtDNA genotype of progeny was identified. Each experiment was performed in more than 32 replicates. Significant differences were determined by performing chi-square tests in Past Statistics, version 3.12. The significance levels are *p < 0.05, **p < 0.01, and ***p < 0.001.

### Fitness competition assay

Two types of hybrids were constructed by crossing *S*. *cerevisiae* with *S*. *paradoxus* (strains W303 × YDG 197 and strains UWOPS83-787.3 × N-17). The halo assay was conducted to determine pheromone response. The hybrid cells were cultured in the YPD medium with daily serial transfer (1:1000) until the pure mtDNA genotype hybrid was obtained. After that, cells were streaked on YPD agar plates and six independent clones of each cross were used in fitness assay.

The hybrid cells were grown in YPD medium for 12 hours. Equal concentrations of the hybrids carrying different mtDNA genotypes was mixed under different conditions with daily serial transfer (1:1000). We measured the fitness difference between the hybrid cells with different mtDNA genotypes cultured under different conditions (5% glucose, glycerol, 0.5 M NaCl chloride, or 100 mM NH_4_Cl) at 28°C. After the same generations in the previous mtDNA inheritance experiment were obtained, the mtDNA genotypes were identified. Each fitness competition assay was conducted in more than 32 replicates.

### Determining mitochondrial activity

The TTC reagent (Sigma, USA) was used to measure dehydrogenase activity. The TTC reagent (5 g TTC dissolved in 500 mL distilled water) was prepared and stored in the dark at 4°C. The solution was diluted 10-fold in sterile ddH_2_O before use. The hybrid cells were grown in YPD medium for 12 hours at 28°C to ensure fresh and healthy cell growth. The hybrid cells were cultured in YPG medium (1:1000) and incubated for 24 hours to ensure that all cells are in respiratory-competent condition. Transferred the cell with same concentration under different conditions and incubated at 28°C for 10–11 hours to ensure that the cells were in the log phase. The cells were then collected at a density of 1 × 10^8^ cell/mL, pelleted, and washed with sterile ddH_2_O. Approximately 500 μL of 0.1% TTC solution was added to the tube and incubated at room temperature for 30 minutes. The supernatant was removed, and the pellet was re-suspended in 800 μL of 95% ethanol to extract the red formazan by mixing for 1 hour on a shaker. The absorbance of the extract was measured at 485 nm by using a spectrophotometer. The assays were performed in nine independent experiments, and the values were expressed as mean ± SD. Comparisons among the experimental groups were made with the paired *t* test (two groups) by using Past Statistics, version 3.12. All P values are two-tailed, and the significance levels were *p < 0.05, **p < 0.01, and ***p < 0.001.

## Results

### Environmental factors result in biased mtDNA transmission in hybrids

*S*. *cerevisiae* and *S*. *paradoxus* were grown and crossed at 28°C to produce diploid hybrids. The hybrid cells were picked and immediately cultured under five different conditions: 2% glucose, 5% glucose, non-fermentable carbon source glycerol-based medium (YPG), 0.5 M NaCl to alter osmotic pressure, and 100 mM NH_4_Cl to inhibit ubiquitination. After hybrids formed colonies on the solid agar plate, all hybrid cells were grown in liquid medium under the respective conditions, and growth was maintained with daily serial transfers (1:1000 dilution). During growth, the total genomic DNA from each tube was extracted, and *COX3* was amplified using a specific primer. The restriction enzymes *Hin*dIII and *Bsm*I was used to identify the mtDNA genotypes, because of the polymorphic sites between *S*. *cerevisiae* and *S*. *paradoxus* (S1 Fig).

Under these conditions, the hybrids were cultured for approximately 110 generations, and the mtDNA genotype was examined every 2 days (~18 generations) (data not shown). In 2% glucose medium, mtDNA inheritance is biased because hybrids with Sp mtDNA overcame those with Sc mtDNA by crossing *S*. *cerevisiae* strain W303 with *S*. *paradoxus* strain YDG 197 and vice versa by crossing *S*. *cerevisiae* strain UWOPS83-787.3 with *S*. *paradoxus* strain N-17 (Tables 1 and 2). When crossing *S*. *cerevisiae* strain W303 with *S*. *paradoxus* strain YDG 197, the results showed that the mtDNA transmission patterns in hybrid progeny under the 5% glucose and osmotic pressure conditions were biased compared with those in the 2% glucose condition. The hybrids under the 5% glucose condition produced hybrid progeny predominantly carrying the *S*. *cerevisiae* mtDNA genotype (p = 7.681 × 10^−5^, χ2 = 18.948, df = 2 compared with those in the 2% glucose condition; Table 1). The hybrids under the osmotic pressure culture condition also produced a high proportion of populations with the *S*. *cerevisiae* mtDNA genotype (p = 9.2563 × 10^−5^, χ2 = 18.575, df = 2 compared with those in the 2% glucose condition; Table 1).

We used different strains to address whether the genetic background contributes the mtDNA transmission patterns in hybrids. When crossing *S*. *cerevisiae* strain UWOPS83-787.3 with *S*. *paradoxus* strain N-17, the results showed that the mtDNA genotype in the hybrids was predominantly inherited from *S*. *cerevisiae* under the glycerol condition (p = 1.1917 × 10^−2^, χ2 = 8.8595, df = 2 compared with those in the 2% glucose condition). By contrast, the hybrids under the 5% glucose, NaCl, and NH_4_Cl culture conditions produced a high proportion of populations with the *S*. *paradoxus* mtDNA genotype (p = 2.5484 × 10^−2^, χ2 = 7.3394, df = 2 compared with those in the 2% glucose condition; p = 1.362 × 10^−2^, χ2 = 8.5924, df = 2 compared with those in the 2% glucose condition; p = 6.4751 × 10^−4^, χ2 = 14.685, df = 2 compared with those in the 2% glucose condition, respectively; Table 2). According to these results, the inheritance of mtDNA was uniparental, but the mtDNA inheritance patterns were environment and strain dependent.

### The effects of fitness difference on biased mtDNA inheritance

**Fig 2:**
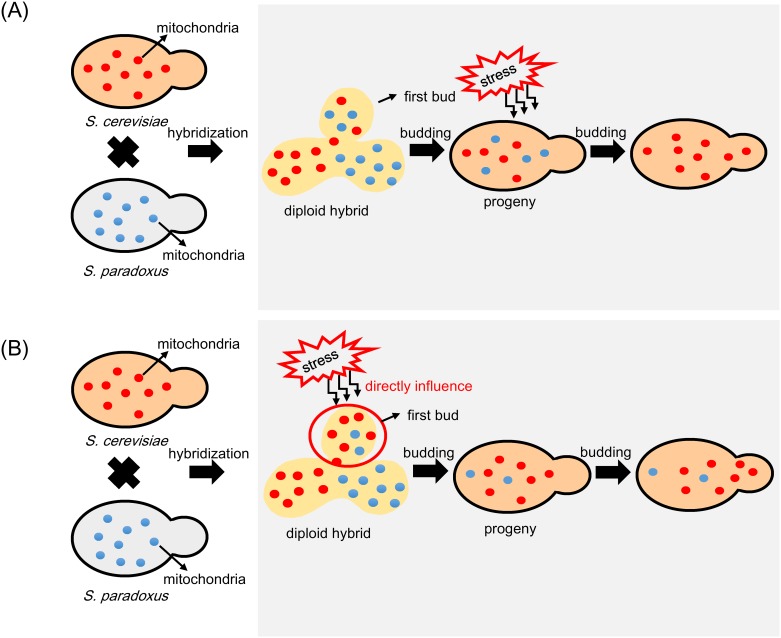
Two possible hypotheses can explain biased mtDNA transmission in yeast hybrids. (A) Fitness differences exist between the hybrids carrying different mtDNA genotypes. After competition, the cells with higher fitness become dominant. (B) Stress influences mtDNA transmission during budding of progeny directly. Under stress, mtDNA transmission to progeny is biased toward one type of mtDNA genotype.

Two possible hypotheses can explain biased mtDNA transmission in hybrids. One possibility for biased mtDNA transmission in hybrids at different stress conditions is that there are fitness differences between the hybrids carrying different mtDNA genotypes. In our results, we observed biased transmission when zygotes were cultured under specific stress conditions compared to 2% glucose environment. Thus, it is possible the mtDNA genotypes of the hybrids were initially mixed mitochondrial genotypes and was randomly fixed in successive generations. However, the hybrids carrying different mtDNA genotypes might show different fitness under stress conditions. After competition, the cells with higher fitness will be the major population and their mtDNA genotype will be detected by our measurement (Fig 2A). Second, stress influences directly on mtDNA transmission during hybrid (or bud) formation. Thus, under stress, mtDNA transmission to progeny is biased toward one type of mtDNA genotype (Fig 2B).

To investigate these possibilities, we generated yeast hybrids with mitochondria derived only from a single parent. After obtaining four types of hybrids [(S. cer W303 × S. par YDG 197) ^ScerMt^ and (S. cer W303 × S. par YDG 197) ^SparMt^; (S. cer UWOPS83-787.3 × S. par N-17) ^ScerMt^ and (S. cer UWOPS83-787.3 × S. par N-17) ^SparMt^] by crossing *S*. *cerevisiae* with *S*. *paradoxus*, we mixed equal concentrations of the hybrids carrying different mtDNA genotypes under different conditions. After obtaining the same generations (~110 generations) in the previous mtDNA inheritance experiment, we identified the mtDNA genotypes. We tested 36 mixed samples under each condition. If biased transmission can be mainly explained by the fitness difference between the hybrids carrying the *S*. *cerevisiae* or *S*. *paradoxus* mtDNA genotype, we expect to obtain the same mtDNA genotype pattern as the previous experiment. In Table 3, the results showed that the hybrids (W303 × YDG 197) ^ScerMt^ had higher fitness than did those carrying *S*. *paradoxus* mtDNA in 5% glucose medium, glycerol-based medium and NH_4_Cl conditions. Only the patterns in 5% glucose medium is the same as that in mtDNA inheritance experiment. It indicates the biased transmission could be explained by the fitness difference between these hybrids in this specific stress. Similar results found in the hybrids (UWOPS83-787.3 × N-17) ^ScerMt^ had higher fitness than did those carrying *S*. *paradoxus* mtDNA in glycerol-based medium. The biased pattern in glycerol-based medium is the same as that in mtDNA inheritance experiment. It again indicates the biased transmission could be explained by the fitness difference between these hybrids in this glycerol-based stress.

**Table 3:** Summary of the results of fitness competition assay and mtDNA inheritance experiments in Tables 1 and 2.

	5% Glucose	Glycerol	5 M NaCl	100 mM NH_4_Cl
**Fitness competition assay**				
*S*. *cerevisiae* W303 x *S*. *paradoxus* YDG 197	Sc	Sc	ScSp	Sc
*S*. *cerevisiae* UWOPS83-787.3 x *S*. *paradoxus* N-17	ScSp	Sc	Sc	ScSp
**mtDNA inheritance experiments**				
*S*. *cerevisiae* W303 x *S*. *paradoxus* YDG 197	Sc	Sp^1^	Sc	Sp and ScSp^1^
*S*. *cerevisiae* UWOPS83-787.3 x *S*. *paradoxus* N-17	Sp	Sc	Sp	Sp

^1^ No significant difference was observed compared with those in the 2% glucose condition.

In contrast, the hybrids (UWOPS83-787.3 × N-17) ^ScerMt^ showed higher fitness under NaCl culture conditions, but mtDNA inheritance was biased toward *S*. *paradoxus* in this stress. From these result, we concluded that the fitness advantage of *S*. *cerevisiae* mtDNA genotype in UWOPS83-787.3 × N-17 hybrids in osmotic stress cannot fully explain for biasd *S*. *paradoxus* mtDNA genotype inheritance in hybrids under this conditions. Similar results were found in the hybrids (W303 × YDG 197) ^ScerMt^ which showed higher fitness under glycerol-based culture conditions, but mtDNA inheritance was biased toward *S*. *paradoxus* in this stress. It indirectly indicates that there is a preference of mitochondrial inheritance of *S*. *paradoxus* mtDNA during hybrids when mating at these stress conditions.

However, in the hybrids produced by crossing W303 with YDG 197 under the NaCl culture condition and those produced by crossing UWOPS83-787.3 with N-17 under the 5% glucose and NH_4_Cl culture conditions, no fitness differences existed between the hybrids carrying the *S*. *cerevisiae* or *S*. *paradoxus* mtDNA genotype, but mtDNA inheritance was biased toward one type of mtDNA genotype in the previous experiment (Table 3). Based on the results, fitness advantage of mtDNA genotype might not play roles in bias mtDNA genotype inheritance in hybrids at these conditions of these hybrids. It again indirectly indicates that there is a preference of mitochondrial inheritance when mating at these conditions in these hybrids.

### Mitochondrial activity in hybrid progeny

After obtaining the results of fitness difference in bias mtDNA inheritance, we determined whether mitochondrial activity influenced the fitness difference between the hybrids carrying the *S*. *cerevisiae* or *S*. *paradoxus* mtDNA genotype under different conditions. Hence, we examined the respiratory competency of the hybrid cells under different conditions by using TTC, which stains respiratory-competent cells red.

**Fig 3:**
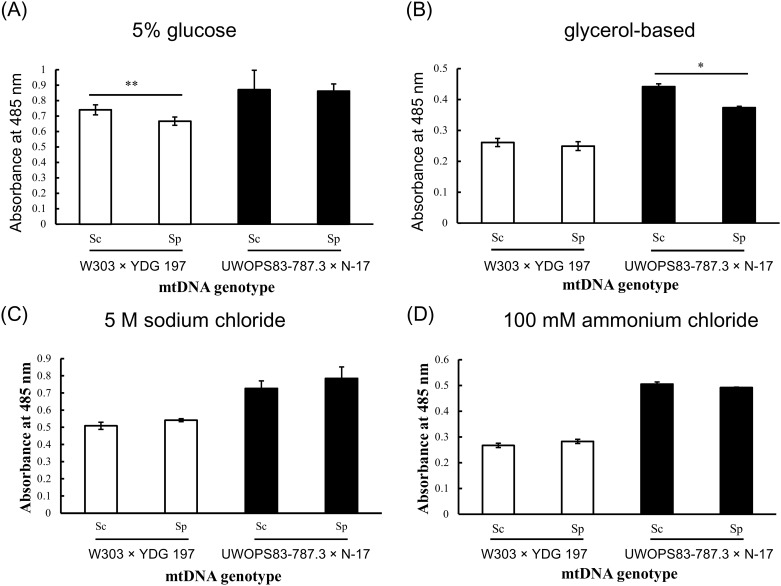
Mitochondrial activity of hybrid cells carrying different mtDNA genotypes cultured in 5% glucose, non-fermentable carbon source glycerol-based medium, 0.5 M NaCl to alter osmotic pressure, and 100 mM NH_4_Cl to inhibit ubiquitination, respectively. (A) Under the 5% glucose condition, the results showed higher mitochondrial activity in the hybrids carrying *S*. *cerevisiae* mtDNA than in those carrying *S*. *paradoxus* mtDNA that were produced by crossing strain W303 with strain YDG 197. However, mitochondrial activity did not significantly differ between the hybrids carrying different mtDNA genotypes produced by crossing UWOPS83-787.3 with N-17. (B) Under the glycerol-based condition, the results showed higher mitochondrial activity in the hybrids carrying *S*. *cerevisiae* mtDNA than in those carrying *S*. *paradoxus* mtDNA that were produced by crossing strain UWOPS83-787.3 with strain N-17. However, mitochondrial activity did not significantly differ between the hybrids carrying different mtDNA genotypes produced by crossing W303 with YDG 197. (C-D) According to the results, neither NaCl nor NH_4_Cl influence mitochondrial activity in hybrids carrying different mtDNA genotypes. Values represent the mean ± SD for nine biological replicates. Two-tailed t tests were performed using Past Statistics, version 3.12. *P* < 0.05 was considered statistically significant. *P < 0.05; **P < 0.01.

Under the 2% glucose culture condition and glycerol culture condition, the results showed that mitochondrial activity did not significantly differ between the hybrids carrying different mtDNA genotypes that were produced by crossing W303 with YDG 197. However, in the hybrids from another cross, that is, UWOPS83-787.3 × N-17, the results revealed higher mitochondrial activity in the hybrids carrying *S*. *cerevisiae* mtDNA than in those carrying *S*. *paradoxus* mtDNA (S2 Fig). Under the 5% glucose condition, the results showed higher mitochondrial activity in the hybrids carrying *S*. *cerevisiae* mtDNA than in those carrying *S*. *paradoxus* mtDNA that were produced by crossing strain W303 with strain YDG 197. However, mitochondrial activity did not significantly differ between the hybrids carrying different mtDNA genotypes produced by crossing UWOPS83-787.3 with N-17 (Fig 3A). Under the glycerol-based condition, the results showed higher mitochondrial activity in the hybrids carrying *S*. *cerevisiae* mtDNA than in those carrying *S*. *paradoxus* mtDNA that were produced by crossing strain UWOPS83-787.3 with strain N-17. However, mitochondrial activity did not significantly differ between the hybrids carrying different mtDNA genotypes produced by crossing W303 with YDG 197 (Fig 3B). According to the results in Fig 3C and 3D, neither NaCl nor NH_4_Cl influence mitochondrial activity in hybrids carrying different mtDNA genotypes.

## Discussion

This study examined whether environmental factors influence mitochondrial inheritance in the *Saccharomyces* yeasts. The results demonstrate that among the five tested environmental factors, the mtDNA transmission patterns are influenced by specific environmental factors and are strain dependent rather than species dependent. To the best of our knowledge, this is the first demonstration that environmental factors can influence mtDNA inheritance in baker’s yeast. Although the detail of mitochondrial transmission remains to be explored, here we suggest that the biased mtDNA transmission under stress conditions in this study is probably associated with some following mechanisms.

Individual yeast cells contain around 30–100 copies of mitochondrial DNA [39], which are inherited from both parents during mating. However, the heteroplasmic state of the progeny is transient because they rapidly become homoplasmic through mitotic segregation, during which new buds receive relatively few copies of mtDNA from mother cells [29, 40]. This restoration might be caused by random choice of an mtDNA template for replication and segregation, which is then quickly fixed in progeny by successive rounds of replication. It has been suggested that the *ori* confers a segregation or replication advantage of the hypersuppressive petite mutants which is inherited uniparentally in crosses to strains with wild-type mtDNA [41]. The difference of *ori* number also have been suggested to explain why *S*. *cerevisiae* mtDNA outcompetes *S*. *bayanus* mtDNA and is preferentially transmitted to the progeny in their hybrids (eight *ori* in S. *cerevisiae* but four in *S*. *bayanus*) [30, 42]. However, *S*. *cerevisiae* and *S*. *paradoxus* resemble each other in the number of *ori* sequences (seven in *S*. *paradoxus*). Thus, the biased mtDNA transmission to the hybrid progeny produced by crossing *S*. *cerevisiae* and *S*. *paradoxus* might not be wholly explained by the difference of *ori* number.

During mating, two haploid yeast cells of opposite mating types fuse to form a diploid zygote. It has been reported that only a small fraction of the mtDNA pool is transferred from the zygote to the bud, and that the position of the bud determines which parental cell contributes its mtDNA [43]. Cells that bud from the mid-point of the zygote inherit mtDNA from both parents, whereas buds forming at either end of the zygote usually contain only one type of parental mtDNA [43, 44]. Thus, these behaviors suggest that the gene controlling the bud position might influence mtDNA inheritance.

In this study, we found that the hybrid cells exhibit the preferential transmission of only one type of parental mtDNA—an inheritance pattern that depends on the strain background. In contrast to nuclear DNA, the replication of mtDNA is not strictly linked to cell cycle, and there is no strict control of mtDNA partitioning during cytokinesis. It is likely that some genetic components control the replication rate or maintenance of mtDNA from both parents. Alternatively, the nuclear components of one parent might dominantly and specifically control the replication machinery or inheritance of its own mtDNA [45, 46]. Previous data have indicated that hybrids between closely related species are usually inviable or sterile, because of the functional incompatibility between genes from different species. Zeyl *et al*. [47] showed that the yeast mitochondrial genome could change functionally and cooperatively along with the nuclear genome in as few as 2000 generations. They also found the incompatibility between nuclear and mitochondrial genomes in closely related species reduced fitness. Thus, it is possible that dominant incompatibility may arise in interspecific hybrids and reduces fitness of hybrids and their offspring. Therefore, we suggest that the biased mtDNA transmission observed in this study is also possibly associated with nuclear—mitochondrial (or mitochondrial—mitochondrial) incompatibility [31].

Here we found that one possible explanation for biased mtDNA transmission in yeast hybrids under stress conditions is due to the fitness differences between the hybrids carrying different mtDNA genotypes. In our results, the hybrids with *S*. *cerevisiae* W303 mtDNA had higher fitness compared with those with *S*. *paradoxus* YDG 197 mtDNA under the 5% glucose, glycerol, and 100 mM NH_4_Cl conditions. However, the hybrids with *S*. *cerevisiae* UWOPS83-787.3 mtDNA had higher fitness compared with those with *S*. *paradoxus* N-17 mtDNA under the glycerol and NaCl conditions. A previous study indicated that greater fitness variation has also been observed among *Cryptococcus neoformans* zygotes in progeny under high temperature than that in normal condition [31]. In evolutionary genetics, a mixture of uniparental and occasionally biparental mtDNA inheritance and recombination might help avoid Muller’s Ratchet of irreversible fitness loss due to mutation accumulation in completely asexual genomes [48, 49]. Therefore, this flexible mtDNA inheritance is particularly advantageous under stress conditions. This finding suggests that these inheritance mechanisms enable yeast to have a more rapid rate of adaptation to different environments.

Finally, ubiquitination is a common pathway involved in the recognition and degradation of subcellular materials. In *Chlamydomonas* and mammals, mitochondria are selectively degraded by proteolytic machinery, whereas ubiquitinated mitochondria are maintained in the progeny [20, 24]. A previous study proposed that during mating in *C*. *neoformans*, mtDNA from the MATα parent might be selectively tagged and destroyed soon after cell fusion, resulting in uniparental inheritance of mtDNA from the MATa parent [25]. However, the ubiquitination inhibitor had no effect on mitochondrial inheritance in the fungus *C*. *neoformans* [31]. However, our results showed that the ubiquitination inhibitor NH_4_Cl can influence mtDNA transmission in yeast hybrids. The results are expected to be changed when different strains are crossed. Thus, selective tagging and degradation may play a role in mitochondrial inheritance in yeasts.

Albertin et al. [50] and Solieri et al. [51] both produced interspecific hybrids between *S*. *cerevisiae* and *S*. *uvarum* species and found that mtDNA showed uniparental inheritance in all hybrids after a given number of generations. However, we showed that, for each crossing, there are still hybrids progeny with mtDNA types from both parents after a given number of generations. In Albertin et al. [50] and Solieri et al. [51], hybrids per cross were recurrent cultured on YPD-agar plates from one colony to a given number of generations. These populations underwent severe bottlenecks during subculturing. Thus, only one mitotype was fixed in this treatment. However, in our study, hybrids were maintained by serial transfer (1:1000) in liquid medium. This may explain the differences between these studies. Furthermore, a loop full of the cell suspensions of our samples (hybrids progeny with mtDNA types from both parents) were streaked out on a YPD-agar plates and the mitotype of each colony was tested. We found that the samples were mixtures, either cells with Sc or Sp mtDNAs after a given number of generations. However, only one mitochondrial marker was used here, we cannot rule out the possibility of recombinants.

## Conclusion

*S*. *cerevisiae* and *S*. *paradoxus* were mated at 28°C to generate diploid hybrid cells, which resemble dumbbells. This study evaluated the effect of several environmental factors on mtDNA inheritance. Our results revealed that mtDNA is uniparentally inherited and that the patterns are influenced by different environmental factors. We also observed that the mtDNA inheritance patterns can be influenced by crossing different strains. However, fitness differences exist between the hybrids carrying different mtDNA genotypes. According to the fitness competition assay results, the fitness advantage of the mtDNA genotype can explain the biased mtDNA genotype inheritance in hybrids cultured under specific stress conditions. In addition, all diploid hybrid cells exhibited respiratory competency. After the TTC assay, we found that fitness differences cannot fully be explained by mitochondrial activity in hybrids under stress conditions.

## Supporting Information

S1 Fig Restriction enzyme digestion to distinguish mtDNA genotype. Total DNA was extracted from each sample, and PCR amplification was performed using the primer flanking mitochondrial *COX3*. PCR products were digested with *Hin*dIII, which cut *S*. *paradoxus COX3*, and *Bsm*I, which cut *S*. *cerevisiae COX3*. The results of enzyme digestion products were verified by agarose gel electrophoresis to distinguish the mtDNA genotype.
(TIF)

S2 Fig Mitochondrial activity of hybrid cells carrying different mtDNA genotypes cultured in YPD medium containing 2% glucose. The results showed that mitochondrial activity did not significantly differ between the hybrids carrying different mtDNA genotypes that were produced by crossing W303 with YDG 197. However, in the hybrids from another cross, that is, UWOPS83-787.3 × N-17, the results revealed higher mitochondrial activity in the hybrids carrying *S*. *cerevisiae* mtDNA than in those carrying *S*. *paradoxus* mtDNA. Values represent the mean ± SD for nine biological replicates. Two-tailed t tests were performed using Past Statistics, version 3.12. *P* < 0.05 was considered statistically significant. *P < 0.05; **P < 0.01.
(TIF)
