# Supplementary figures and images for: Environmental Factors Can Influence Mitochondrial Inheritance in the Saccharomyces Yeast Hybrids
Source: PLoS One. 2017 Jan 12;12(1):e0169953. doi: 10.1371/journal.pone.0169953 (PMC5231273; doi:10.1371/journal.pone.0169953)

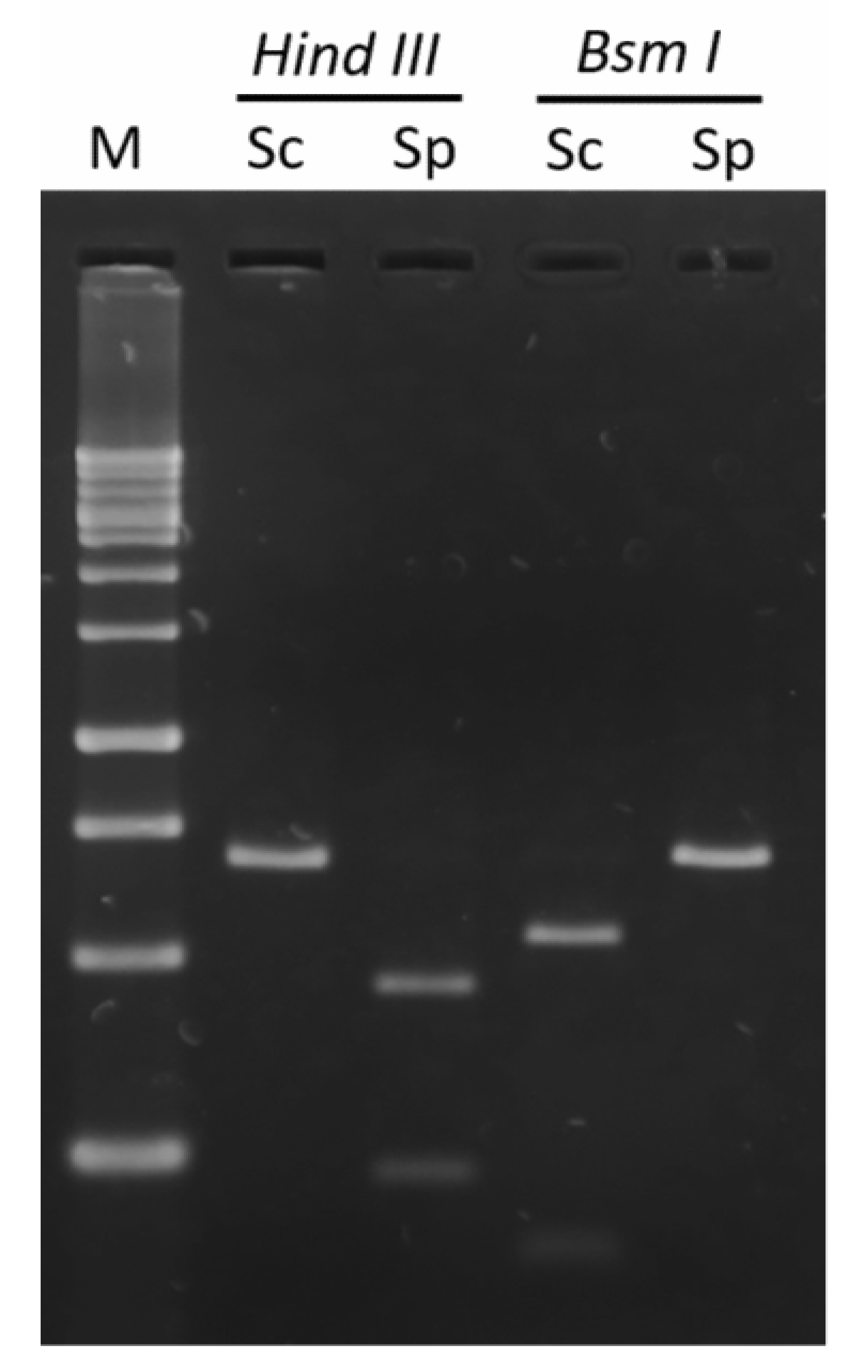

Supplement: S1 Fig — Total DNA was extracted from each sample, and PCR amplification was performed using the primer flanking mitochondrial COX3. PCR products were digested with HindIII, which cut S. paradoxus COX3, and BsmI, which cut S. cerevisiae COX3. The results of enzyme digestion products were verified by agarose gel electrophoresis to distinguish the mtDNA genotype. (TIF) [file pone.0169953.s001.tif]

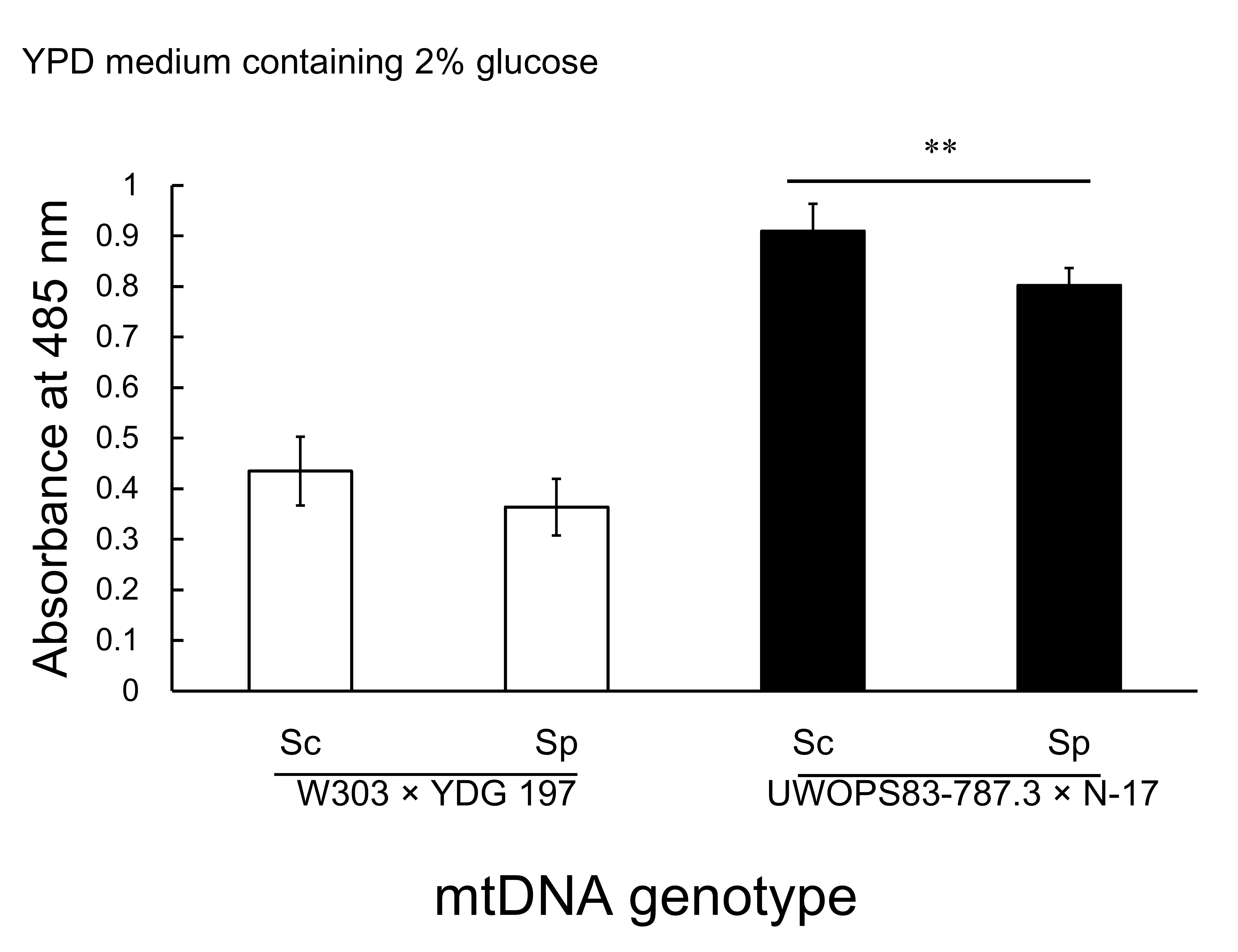

Supplement: S2 Fig — The results showed that mitochondrial activity did not significantly differ between the hybrids carrying different mtDNA genotypes that were produced by crossing W303 with YDG 197. However, in the hybrids from another cross, that is, UWOPS83-787.3 × N-17, the results revealed higher mitochondrial activity in the hybrids carrying S. cerevisiae mtDNA than in those carrying S. paradoxus mtDNA. Values represent the mean ± SD for nine biological replicates. Two-tailed t tests were performed using Past Statistics, version 3.12. P < 0.05 was considered statistically significant. *P < 0.05; **P < 0.01. (TIF) [file pone.0169953.s002.tif]
